# Five-Year outcomes of a digitally delivered carbohydrate-reduced nutrition intervention for prediabetes: durability of diabetes prevention

**DOI:** 10.3389/fnut.2026.1839029

**Published:** 2026-06-19

**Authors:** Alison R. Zoller, Shaminie J. Athinarayanan, Michelle R. Van Tieghem, Rebecca N. Adams, Caroline G. P. Roberts, Jeff S. Volek, Stephen D. Phinney, Amy L. McKenzie

**Affiliations:** 1 Virta Health, Denver, CO, United States; 2 Genesis Research, Hoboken, NJ, United States; 3 Department of Human Sciences, The Ohio State University, Columbus, OH, United States; 4 School of Medicine, University of California, Davis, Davis, CA, United States; 5 Abbott, Lingo Germany Gmbh, Wiesbaden, Germany

**Keywords:** disease prevention, low carbohydrate, metabolic disease, normoglycemia, prediabetes, remote continuous care, telemedicine

## Abstract

**Introduction:**

Sustained prevention of type 2 diabetes (T2D) remains a major public health challenge, as benefits from traditional lifestyle and earlier pharmacologic interventions have often been difficult to maintain. Prediabetes is associated with increased cardiometabolic disease risk and mortality even without progression to diabetes. Interventions that achieve long-term metabolic health improvements are needed. This study reports five-year outcomes of a continuous remote care intervention (CCI) delivering individualized carbohydrate-reduced nutrition therapy in adults with prediabetes.

**Methods:**

A three-year prospective, single-arm longitudinal study extended a previously reported two-year trial (ClinicalTrials.gov NCT02519309). Adults 21–65 years with prediabetes were enrolled (*n* = 96) in the original two-year trial; 58 participants consented to a three-year extension. Outcomes assessed over 5 years included glycemic status, weight, insulin resistance, lipids, inflammatory markers, and liver and kidney function. Longitudinal changes were analyzed using generalized estimating equations and linear mixed-effects models as part of an extension cohort analysis.

**Results:**

At five years, 45 participants (47% of the original cohort) completed follow-up with 22% achieving normoglycemia (*P* < 0.001), and 13% progressing to T2D (*P* < 0.01). The cumulative incidence (i.e., achievement at any time point) of regression to normoglycemia was 55.6%, and progression to T2D was 12.2% over five years. Fasting insulin, HOMA-IR, triglycerides, HDL-C, LDL-C, non-HDL-C, and hsCRP were maintained at or below baseline levels at five years; however, these differences were not statistically significant after adjustment, despite unadjusted *P*-values ≤ 0.05. Mean body weight was reduced by 5.8 kg (5.3%; *P* < 0.001), with 47%, 29%, and 13% achieving ≥5%, ≥10%, and ≥15% weight loss, respectively. Liver and kidney function was stable.

**Conclusion:**

Participation in a continuous remote care model delivering individualized carbohydrate-reduced nutrition therapy was associated with significant weight reduction and maintenance of glycemic status, insulin resistance, and cardiometabolic risk markers over five years among adults with prediabetes. Although mean glycemic markers were comparable to baseline at five years, a substantial proportion of participants achieved normoglycemia and relatively few progressed to T2D, suggesting potential for long-term diabetes prevention with a digitally supported, nutrition-focused intervention.

## 1 Introduction

Sustained diabetes prevention remains an unmet public health challenge. Globally, over 460 million people have prediabetes with prevalence projected to increase substantially in the coming decades (1). In the United States, more than one-third of adults—approximately 98 million people—have prediabetes (2), and without intervention, up to 70% will eventually progress to type 2 diabetes (T2D) (3). Even before progression, prediabetes is associated with elevated risk for cardiovascular disease, chronic kidney disease, and all-cause mortality (4–6), underscoring the need for interventions that not only prevent diabetes onset but also durably maintain metabolic health improvements over the long term.

Traditional diabetes prevention programs achieve early success but rarely sustain it. The Diabetes Prevention Program (DPP) reduced diabetes incidence by 58% over 3 years through modest weight loss and increased physical activity. Despite these early successes, subsequent follow-up demonstrated limited durability of benefit. In the Diabetes Prevention Program Outcomes Study (DPPOS), diabetes prevention effects diminished over time in the context of substantial weight regain, with more than half of participants ultimately developing diabetes (7). This lack of long-term weight maintenance is consistent with findings from other lifestyle and dietary interventions, including low-calorie and low-fat approaches (8). Even pharmacologic approaches that produce substantial metabolic improvements—such as GLP-1 receptor agonists—show a regression of benefits upon discontinuation (9). Recognizing these challenges, the American Diabetes Association emphasizes the need for durable lifestyle modification and individualized nutrition therapy for diabetes prevention, noting that low-carbohydrate eating patterns can be an effective option when adherence is maintained over time (10). Collectively, these findings underscore a persistent limitation of existing approaches—the capacity to translate early metabolic change into long-term maintenance.

A continuous remote care intervention (CCI) was developed to address this durability gap in diabetes prevention by pairing an effective nutrition strategy with continuous, app-enabled clinical and behavioral support. The CCI integrates individualized low-carbohydrate nutrition therapy with ongoing clinician supervision, biomarker feedback, and digital health coaching to promote sustained adherence and accountability over time. In a two-year pilot of this model among adults with prediabetes, we reported substantial improvements in glycemia, body weight, and cardiometabolic risk markers, with more than half of participants achieving reversion to normoglycemia and very low rates of progression to diabetes (11). In adults with established T2D, a three-year extension demonstrated durable five-year improvements in similar metabolic health markers (12–14), providing strong evidence that both metabolic and behavioral sustainability are achievable. However, comparable long-term data in adults with prediabetes remain limited, motivating the present five-year assessment of durability and sustainability of this approach.

The present study therefore extends our prior two-year findings by reporting five-year outcomes of a CCI with low-carbohydrate nutrition therapy in adults with prediabetes. We assessed changes in the proportion of participants who regressed to normoglycemia or progressed to diabetes and evaluated long-term changes in glycemic control, body weight, and cardiometabolic health markers—providing rare evidence of the durability and sustainability of a scalable, nutrition-based diabetes prevention program.

## 2 Methods

### 2.1 Study design and participants

**Figure 1:**
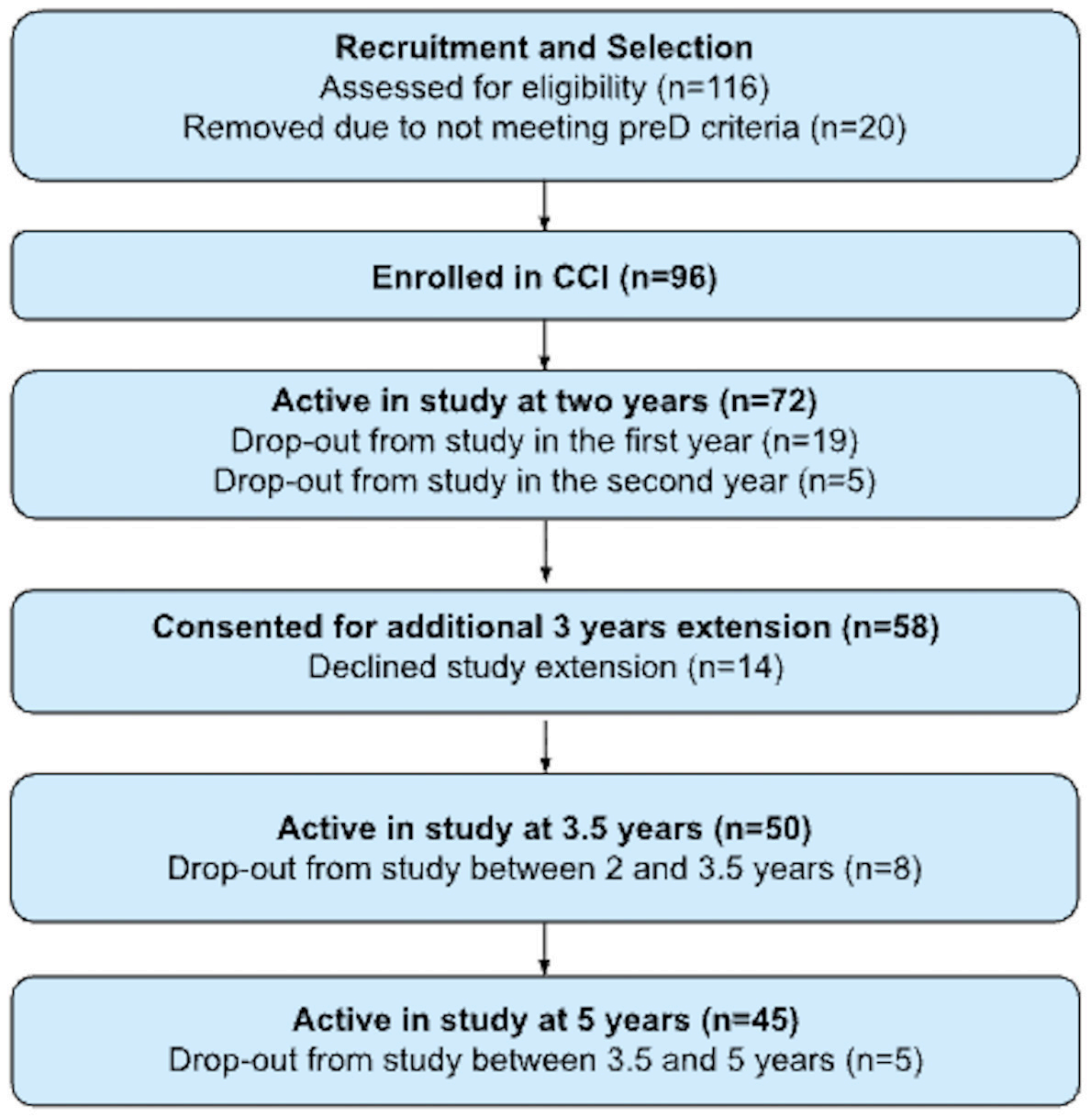
Flowchart of participants in the continuous care intervention (CCI) from baseline to 5 years.

The initial cohort enrolled in a two-year, prospective, single-arm, longitudinal pilot study (Clinicaltrials.gov Identifier NCT02519309) to assess the effects of a CCI developed and delivered by Virta Health for T2D prevention (11). This manuscript reports on the three-year extension of that study, in which we evaluated the long-term metabolic health effects of the CCI among participants with prediabetes. The initial pilot recruited participants (*n* = 96) between August 2015 and March 2016 from Lafayette, Indiana and surrounding areas, and aimed to normalize glycemia and improve metabolic health using a very low-carbohydrate nutrition intervention. Details of recruitment, informed consent, and inclusion and exclusion criteria were previously published (11, 12) with eligibility criteria including adults aged 21 to 65 years with a medical diagnosis of prediabetes and features of metabolic syndrome (exclusions included advanced renal/hepatic/cardiac dysfunction, dietary fat intolerance, and pregnancy/planned pregnancy). We invited the 72 of 96 (75%) participants who completed the initial two-year study to enroll in a three-year extension (Figure 1). The 58 of 72 participants who re-consented (81%) continued to receive support through a web-based platform, which allowed remote monitoring and interaction with a care team consisting of a health coach and a medical provider. We did not require additional in-person classes or visits, though optional clinic-based visits were available upon request and participants met with the study physician for laboratory reviews after outcome assessments at 3.5 and 5 years. The Franciscan Health Lafayette Institutional Review Board approved all study procedures; all participants provided written informed consent at initial enrollment and re-consented for the three-year extension.

### 2.2 Intervention

We delivered the CCI through a mobile web-based application that facilitated remote care by connecting participants with a multidisciplinary care team, including a health coach for behavior and nutrition support and a medical provider who oversaw biomarker monitoring and medication management. Participants collected and recorded their biomarker data for adherence monitoring—including fingerstick blood glucose and beta-hydroxybutyrate (BHB; Precision Xtra, Abbott), and weight (BT003, BodyTrace)—directly into the app. We initially instructed participants to record weight and BHB daily and measure blood glucose 1–3 times per day; we later individualized recording frequency based on participant progress and clinical stability. Participants also had ongoing access to educational resources and an app-based peer support community. The nutritional component of the intervention has been detailed extensively (12, 13, 15). In short, we initially recommended intake of a very low carbohydrate diet at a level to promote nutritional ketosis, defined as a blood BHB level of ≥0.5 mmol/L. Initial guidance included limiting carbohydrate intake to fewer than 30 grams per day, consuming 1.5 grams of protein per kilogram of reference body weight per day, and eating dietary fat to satiety. We tailored food intake to individual preferences and tolerances and adjusted BHB recommendations over time based on participants' health status and goals.

### 2.3 Assessments

We conducted clinical outcome measures at baseline and at 1-, 2-, 3.5-, and 5-year time points with baseline, 1-, and 2-year assessments previously detailed (11).

Laboratory blood tests included Hemoglobin A1c (HbA1c), fasting glucose, fasting insulin, a lipid panel, apolipoprotein assessment, high-sensitivity C-reactive protein (hsCRP), white blood cells, kidney function, and liver enzymes and were performed using a Clinical Laboratory Improvement Amendments (CLIA) certified laboratory. We measured anthropometrics (body weight, height) and, where there were CLIA-derived weights missing, app-recorded values within ±15 days of lab draws were substituted. We calculated homeostatic model assessment of insulin resistance (HOMA-IR) from fasting serum insulin and glucose (16). We calculated estimated glomerular filtration rate (eGFR) using the most recent Chronic Kidney Disease Epidemiology Collaboration (CKD-EPI) (17) and LDL-cholesterol (LDL-C) using the National Institutes of Health NIH 2020 equation (18).

We classified glycemic status into dichotomous outcomes based on the following criteria: normoglycemia was defined as an HbA1c below 5.7% without the use of any glycemic control medications including metformin; prediabetes was defined as either an HbA1c between 5.7% and 6.4% (inclusive), or an HbA1c below 6.5% in individuals using metformin; and T2D was defined as an HbA1c of 6.5% or higher regardless of medication use, or an HbA1c below 6.5% in individuals using glycemic medications other than metformin.

### 2.4 Statistical analysis

We summarized baseline and two-year characteristics using unadjusted means and percentages. To compare groups, independent samples *t*-tests were used to assess differences in: 1) baseline and two-year measures between participants who agreed to the three-year study extension and those who declined, and 2) baseline characteristics between individuals who completed 5 years of follow-up and those lost to follow-up between years two and five. Among study completers, mean app-entered BHB values were calculated for each study year as a proxy of adherence to the dietary intervention.

For the dichotomous outcomes, we used generalized estimating equations (GEE) with binary logistic models and an unstructured covariance matrix to evaluate changes over time. GEE accounts for within-subject correlations from repeated measures, providing robust population-averaged estimates. Missing data were not imputed for the primary analysis. We also performed a sensitivity analysis using multiple imputation (100 imputed datasets) to assess the consistency of our findings.

We calculated crude incidence rates for first diagnosis of T2D and attainment of normoglycemia, expressed per 100 person-years. The cumulative incidence of both outcomes over 5 years was estimated using the Kaplan–Meier method.

To evaluate changes from baseline to 5 years in continuous markers of cardiometabolic health (HbA1c, fasting glucose and insulin, HOMA-IR, weight, total cholesterol, HDL-cholesterol, LDL-cholesterol, non HDL-cholesterol, triglycerides, Apolipoprotein B, Apolipoprotein A1, hsCRP, white blood cells, blood urea nitrogen, creatinine, eGFR, bilirubin, aspartate aminotransferase, alanine aminotransferase, and alkaline phosphatase), we used linear mixed-effects models with an unstructured covariance matrix to account for repeated outcomes. For variables showing non-normal distributions, extreme outliers (top 1% of values) were excluded to reduce skew. These models estimated cumulative changes from baseline to 5 years and included time as a fixed effect and baseline age, sex, race, and metformin use as covariates. We analyzed outcomes including HbA1c, weight, fasting insulin, total cholesterol, LDL-C, HDL-C, triglycerides, and non-HDL-C at five time points: baseline, 1, 2, 3.5, and 5 years. We assessed all other continuous outcomes at four time points: baseline, 1, 2, and 5 years. The primary analysis was conducted as an extension cohort analysis, including all participants who consented to the extension phase (*n* = 58). Analyses used maximum likelihood estimation under the assumption that data were missing at random.

Normality of continuous variables was assessed using skewness and kurtosis, with departures from normality defined as values exceeding ± 3 for skewness and ± 10 for kurtosis. Several sensitivity analyses were conducted to evaluate the robustness of the findings to different assumptions. Skewed variables were assessed using three approaches: exclusion of the top 1% of outliers, analysis of the full cohort, and log transformation (with the latter two presented in Supplementary Table 1). To evaluate the potential impact of missing data, including departures from a missing-at-random assumption, simplified pattern-mixture models were performed for HbA1c, weight, and fasting insulin. Given the modest sample size, a simplified approach was used to ensure model stability and interpretability. Participants were classified as completers vs. non-completers based on availability of each outcome at the final time point. Linear mixed-effects models included time, missing-data pattern, and their interaction. Additionally, we conducted a sensitivity analysis of key metabolic outcomes (i.e., HbA1c, fasting glucose, fasting insulin, HOMA-IR, weight) limited to participants who completed the full five-year follow-up with available data; results were similar and therefore are not reported here but available in Supplementary Table 2.

Changes in body mass index (BMI) categories at 5 years compared to baseline, as well as the proportion of participants achieving clinically meaningful weight loss thresholds (e.g., ≥5%, ≥10%, and ≥15% weight loss), were assessed descriptively among participants who completed the five-year follow-up.

For continuous outcomes, unadjusted *P*-values are reported for all time points. To account for multiple comparisons across biomarkers, *P*-values for the five-year time point were additionally adjusted using the Holm-Bonferroni sequential correction method. Statistical significance was defined as a *P*-value less than 0.05. All statistical analyses were conducted using IBM SPSS Statistics, version 28.0 (IBM Corp., Armonk, NY, USA).

## 3 Results

### 3.1 Participant characteristics and retention

Baseline characteristics of those who consented to the long-term follow-up did not differ significantly from those who declined (Table 1), however significant differences at 2 years included HbA1c being lower and BHB levels being higher for the consenters (Supplementary Table 3). For consenters, mean age at enrollment was 53 (8), 83% female, and 5% African American. Mean weight was 109.4 kg (19), with a BMI of 38.9 (7.6) kg/m^2^, and mean HbA1c of 5.9% (0.2).

**Table 1:** Baseline demographic and clinical characteristics of the original study participants by consent decision to the 3-year extension study.

Variables	Consented to 3-year Extension (*n* = 58)	Declined 3-year Extension (*n* = 14)	*P*-value
Demographics	
Age (years)	53.4 (8.3)	48.6 (9.2)	NS
Female (%)	83.0	79.0	NS
African American (%)	5.0	7.0	NS
Clinical characteristics	
Weight (kg)	109.4 (24.0)	103.6 (21.3)	NS
HbA1c (%)	5.9 (0.2)	5.9 (0.2)	NS
Fasting glucose (mg/dl)	111.7 (16.8)	107.2 (7.6)	NS
Fasting insulin (mIU/L)	25.3 (24.1)	23.5 (17.0)	NS
Triglycerides (mg/dl)	159.0 (84.9)	132.6 (28.6)	NS
Total cholesterol (mg/dl)	203.7 (34.4)	200.9 (38.3)	NS
LDL–cholesterol (mg/dl)	124.7 (33.4)	130.3 (32.3)	NS
HDL–cholesterol (mg/dl)	50.8 (14.3)	46.7 (9.7)	NS
Non–HDL–cholesterol (mg/dl)	152.9 (37.0)	154.2 (33.6)	NS
Apolipoprotein B (mg/dl)	107.2 (25.0)	109.6 (21.4)	NS
Apolipoprotein A1 (mg/dl)	163.2 (24.6)	151.9 (22.9)	NS
hs C–reactive protein (nmol/L)	6.9 (5.8)	10.4 (11.3)	NS
White blood cell (k/cumm)	7.0 (1.8)	7.1 (1.4)	NS
ALT (U/L)	28.8 (22.1)	28.1 (14.0)	NS
AST (U/L)	24.1 (18.1)	19.5 (7.0)	NS
ALP (U/L)	73.5 (19.8)	66.2 (16.3)	NS
Bilirubin (mg/dl)	0.5 (0.2)	0.4 (0.1)	NS
BUN (mg/dl)	16.3 (4.6)	15.1 (3.1)	NS
Creatinine (mg/dl)	0.9 (0.2)	0.8 (0.2)	NS
eGFR (mL/min/1.73m2)	79.5 (13.8)	84.1 (8.5)	NS
Beta–hydroxybutyrate (mM)	0.1 (0.1)	0.2 (0.1)	NS

Values are mean (SD) or percentage.

NS, not significant; HbA1c, Hemoglobin A1c; LDL, low density lipoprotein; HDL, high density lipoprotein; ALT, alanine aminotransferase; AST, aspartate aminotransferase; ALP, Alkaline phosphatase; BUN, blood urea nitrogen; eGFR, Estimated glomerular filtration rate.

At three and a half years, 50 of 58 (86%) remained active and 45 of 58 (78%) completed the full 5 years of follow-up (representing 47% of the original cohort). Differences in baseline characteristics between the 45 who completed 5 years of the study and those that were lost to follow up included higher total, LDL, and non-HDL cholesterol and apolipoprotein B (Supplementary Table 4). Among the 45 who completed the study, metformin was prescribed to 10 at baseline and 14 at 5 years. Mean app-entered BHB levels were 0.6, 0.5, 0.5, 0.4, and 0.4 mmol/L for years one through five, respectively. Across the five-years, mean BHB values for individual participants ranged from 0.2 to 1.5 mmol/L, with the range for year four being 0.1 to 1.6 mml/L and for year five being 0.1 to 1.0 mmol/L.

### 3.2 Prevalence and Incidence of diabetes status

**Figure 2:**
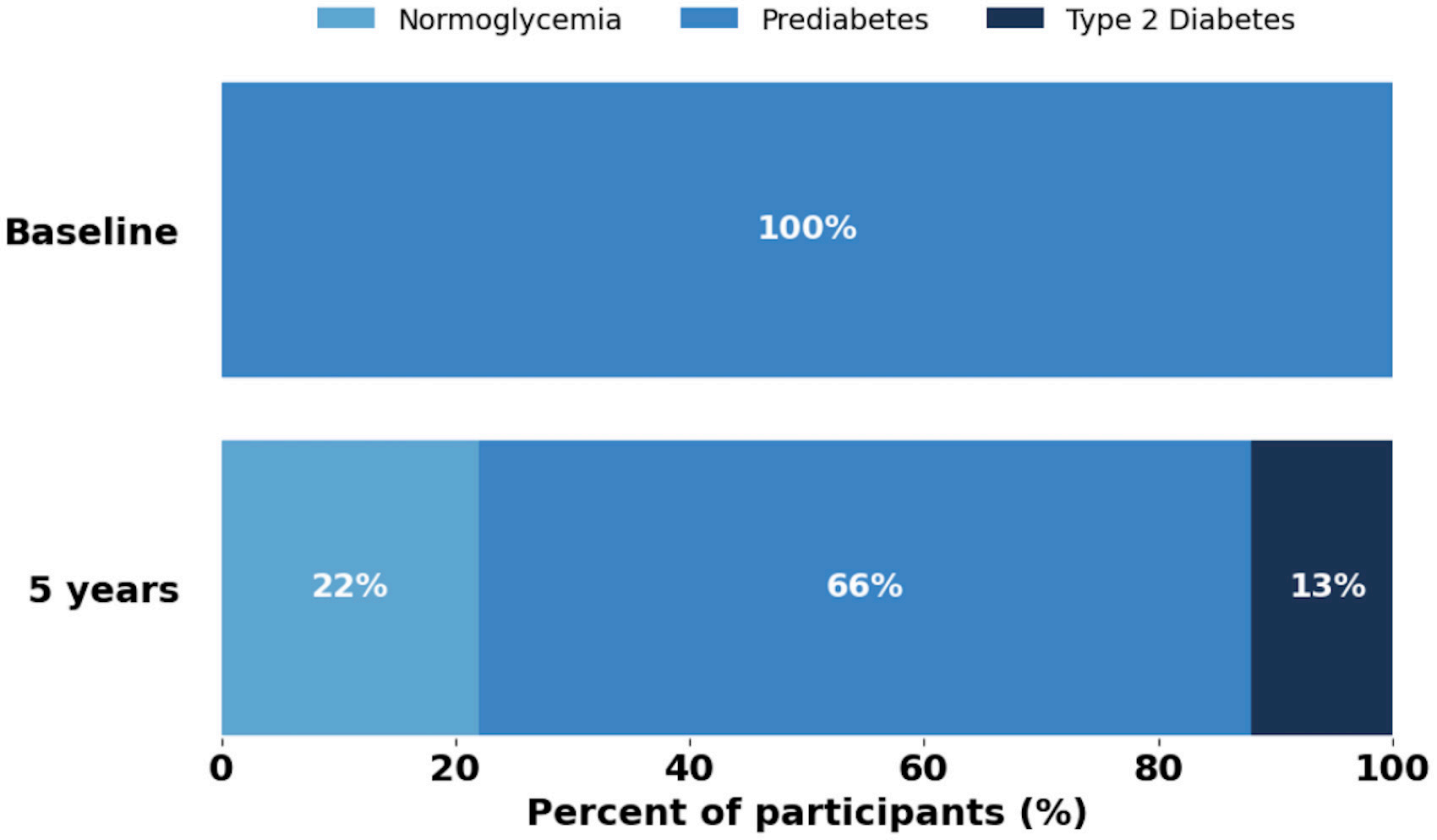
Prevalence of prediabetes, normoglycemia, and type 2 diabetes at baseline and 5 years.

**Figure 3:**
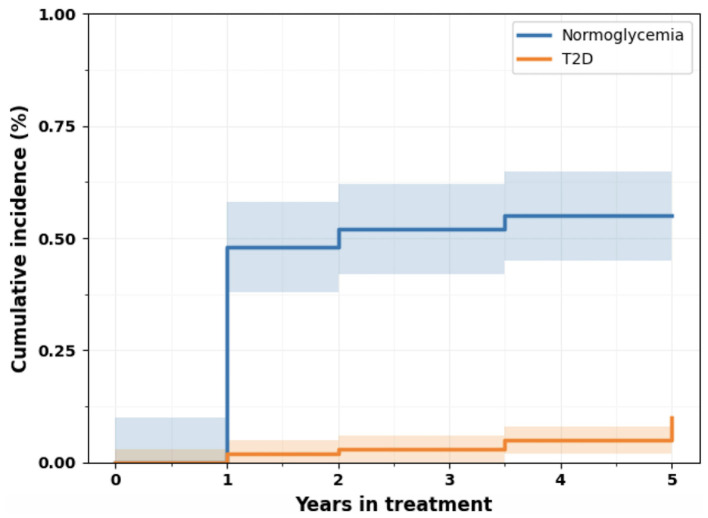
Cumulative incidence of regression to normoglycemia and progression to type 2 diabetes over 5 years (*N* = 58).

Over 5 years, the prevalence of prediabetes declined from 100% at baseline to 66% (SE 7.1% *P* < 0.001). This reduction reflected both regression to normoglycemia, observed in 22% of participants (SE 6.4%; *P* < 0.001), and progression to T2D in 13% (SE 5.5%; *P* < 0.01) by year 5 (Figure 2). Three participants had HbA1c values < 5.7% at 5 years while using metformin and were therefore classified as having prediabetes. Most regression to normoglycemia occurred during the 1 year, while the few cases of progression to T2D (*n* = 6) were distributed relatively evenly across the 5 years (Figure 3). The cumulative incidences of normoglycemia and T2D were 55.6% and 12.2%, respectively.

The estimated crude incidence rate of the first occurrence of regression to normoglycemia was 18.9 per 100 person-years and of T2D was 2.0 per 100 person-years.

### 3.3 Markers of cardiometabolic health

Mean HbA1c decreased after initiation of the intervention, regressed back but remained comparable to baseline level at 5 years (Table 2). Fasting glucose followed a similar pattern, showing early improvement with slight rebound but no significant difference from baseline at year five. In contrast, fasting insulin and HOMA-IR remained lower than baseline at 5 years (unadjusted *P* ≤ 0.05).

**Table 2:** Changes in glycemic, lipid, inflammation, hepatic, and renal markers over time (*N* = 58).

Metabolic markers	Baseline		1 year		2 years		3.5 years		5 years		5 Year Adjusted *P*-value
HbA1c, %	5.9 (0.0)		5.6 (0.0)^***^		5.7 (0.0)^***^		5.8 (0.1)		5.9 (0.1)		NS
Fasting Glucose, mg/dl	111.7 (2.0)		100.5 (1.6)^***^		101.0 (1.7)^***^		–		107.7 (2.5)		NS
Fasting Insulin, mU/L†	22.2 (1.6)		13.5 (1.1)^***^		14.3 (1.2)^***^		16.3 (1.3)^***^		17.5 (1.6)^*^		NS
HOMA–IR†	6.1 (0.5)		3.3 (0.3)^***^		3.7 (0.4)^***^		–		4.7 (0.5)^*^		NS
Weight, kg	109.2 (3.0)		95.0 (2.4)^***^		95.8 (2.4)^***^		100.8 (2.6)^***^		103.4 (2.7)^***^		0.007
TC, mg/dl	203.3 (4.3)		219.0 (6.9)^*^		212.8 (6.2)		200.1 (4.8)		195.6 (4.8)		NS
Triglycerides, mg/dl†	158.4 (11.0)		120.9 (9.4)^***^		114.1 (7.8)^***^		125.2 (8.4)^**^		134.3 (10.9)^*^		NS
HDL–C, mg/dl	50.7 (1.7)		57.1 (2.1)^***^		58.3 (2.3)^***^		55.8 (1.9)^**^		55.7 (2.0)^**^		NS
LDL–C, mg/dl	124.7 (4.2)		140.3 (6.8)^*^		133.7 (5.6)		117.2 (4.8)		115.6 (4.6)^*^		NS
NonHDL–C, mg/dl	152.9 (4.6)		161.6 (6.9)		154.2 (6.5)		144.1 (5.0)		139.3 (4.7)^*^		NS
ApoB, mg/dl	107.1 (3.1)		114.6 (4.4)		105.8 (3.9)		–		103.9 (3.2)		NS
ApoA1, mg/dl	163.8 (2.9)		173.5 (3.9)^**^		174.1 (4.5)^**^		–		160.6 (3.7)		NS
hsCRP, mg/L†	6.8 (0.7)		5.3 (0.8)^**^		5.0 (0.8)^**^		–		4.4 (0.5)^**^		NS
WBC, k/mm3	7.0 (0.2)		6.4 (0.2)^***^		6.4 (0.2)^***^		–		6.3 (0.3)^***^		0.02
BUN, mg/dl	16.3 (0.5)		17.4 (0.7)		16.4 (0.7)		–		16.7 (0.9)		NS
Creatinine, mg/dl†	0.9 (0.0)		0.8 (0.0)^***^		0.8 (0.0)^***^		–		0.8 (0.0)^*^		NS
GFR, ml/min/1.73m2	79.4 (1.7)		82.8 (1.4)^**^		82.2 (1.7)^*^		–		80.5 (2.2)		NS
Total Bilirubin, mg/dl	0.5 (0.0)		0.5 (0.0)		0.5 (0.0)		–		0.4 (0.0)^*^		NS
AST, IU/L†	22.1 (1.2)		19.9 (0.6)		20.3 (0.7)		–		22.5 (1.1)		NS
ALT, IU/L†	25.8 (1.6)		21.8 (1.1)^**^		22.1 (1.2)^*^		–		27.3 (2.0)		NS
ALP, IU/L	73.4 (2.4)		66.0 (2.1)^**^		68.0 (1.9)^*^		–		84.7 (3.0)^***^		0.01

Values are mean (SE). Estimated means and standard errors were derived from linear mixed effects models with an unstructured covariance matrix to account for repeated outcomes using the maximum likelihood approach. Covariates included in the models were baseline age, sex, race, and metformin use. † Top 1% of values omitted. All P-values are compared to baseline. ^*^ P < 0.05, ^**^ P < 0.01, ^***^ P < 0.001, NS not significant. Adjusted P-values for year 5 used the Holm-Bonferroni sequential correction method. “–” lab marker not collected at this time point.

HbA1c, Hemoglobin A1c; HOMA-IR, homeostatic model assessment for insulin resistance; TC, total cholesterol; LDL-C, low density lipoprotein cholesterol; HDL-C, high density lipoprotein cholesterol; hsCRP, Highly Sensitive C-Reactive Protein; WBC, White Blood Cell; BUN, Blood Urea Nitrogen; GFR, Glomerular Filtration Rate; AST, Aspartate Aminotransferase; ALT, Alanine Aminotransferase; ALP, Alkaline Phosphatase.

**Figure 4:**
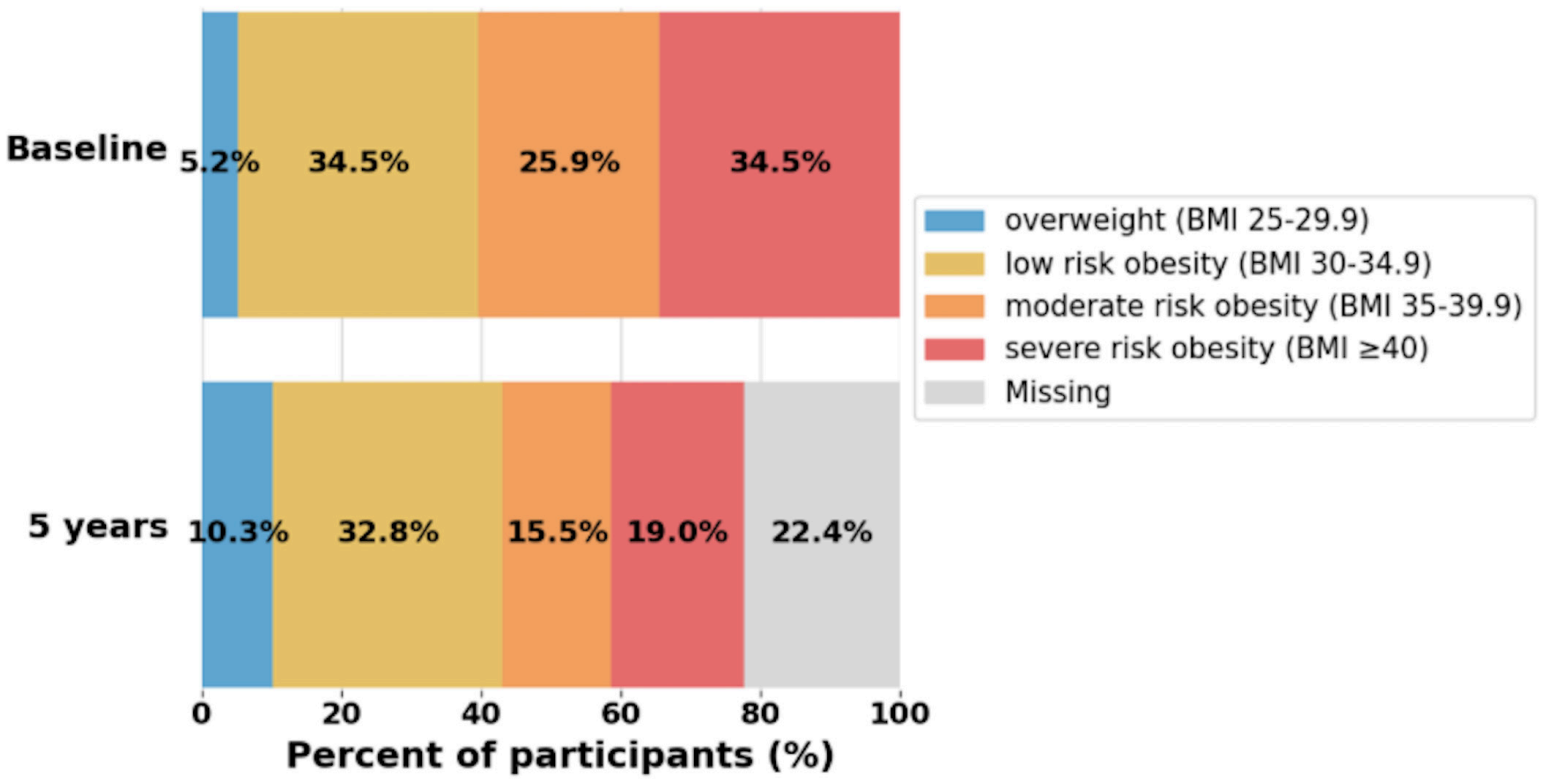
Prevalence of obesity classes and body mass index categories among participants at baseline and 5 years.

At 5 years, participants maintained a significant reduction in body weight compared with baseline (−5.8 kg; *P* < 0.01). At 5 years, 47%, 29%, and 13% achieved ≥5%, ≥10%, and ≥15% weight loss, respectively. The distribution of BMI categories shifted toward lower risk classifications, with the proportion of participants classified as having a BMI ≥35 kg/m^2^ decreasing from 60.4% to 34.5%, while those classified as overweight (BMI < 30 kg/m^2^) nearly doubled (Figure 4). The weight loss achieved early in the study regressed somewhat but at 5 years remained 5.3% below baseline (*P* < 0.01).

Lipid profiles remained lower than baseline at 5 years, with improvements (unadjusted *P* values < 0.05) observed in triglycerides, HDL-C, LDL-C, and non–HDL-C (Table 2). hsCRP significantly decreased in unadjusted analyses (*P* < 0.01), although this was not statistically significant after adjustment. Liver and kidney markers remained within normal limits, with stable eGFR, serum creatinine, and total bilirubin. From baseline to 5 years, there were no significant changes in AST and ALT, and both markers were within the normal range throughout the study.

In simplified pattern-mixture models, the inclusion of missing-data pattern and time-by-pattern interaction terms did not materially change the estimated longitudinal trends in HbA1c, weight, or fasting insulin. However, given the limited sample size and potential for reduced statistical power, these analyses may not be sufficient to detect departures from the missing-at-random assumption. Accordingly, these findings should be interpreted cautiously and do not definitively establish robustness to potential bias from missing data.

## 4 Discussion

These results from this study of low carbohydrate nutrition therapy for prediabetes demonstrate sustained reversion of prediabetes to normoglycemia and prevention of T2D progression among most participants enrolled five-years. With a 47% retention rate at 5 years from the original cohort, participants experienced low rates of progression to T2D, maintained clinically significant weight loss, and improved multiple cardiometabolic risk markers. These findings indicate that a personalized, digitally supported, continuous remote care model may support long-term prevention of diabetes.

At 5 years, 88% of participants had not progressed to T2D, with 22% achieving normoglycemia and 66% remaining in the prediabetes range. Consistent with these findings, the cumulative incidence of regression to normoglycemia was 55.6%, while only 12.2% progressed to T2D over 5 years. These outcomes compare favorably with long-term outcomes reported for the DPP Lifestyle group, in which approximately 24% of participants progressed to diabetes by 5 years following attenuation of early benefit despite intensive, in-person support (19, 20). Other diabetes prevention trials have also reported early regression to normoglycemia (21, 22); however, sustained prevention over multiple years remains uncommon. Importantly, we also observed sustained reductions in fasting insulin out to 5 years, which corresponds with improvements in HOMA-IR and indicates durable improvements in insulin sensitivity.

In our cohort, the durability of normoglycemia observed at 5 years represents a meaningful clinical achievement, reflecting sustained metabolic improvement at the individual level. Notably, this categorical improvement occurred despite mean fasting glucose and HbA1c values at 5 years being comparable to baseline values. This discrepancy likely reflects heterogeneity in individual glycemic outcomes with the many participants regressing to normoglycemia being offset by a smaller number who progressed to T2D. As a result, mean glycemic values may obscure clinically meaningful shifts in glycemic status distribution. These findings highlight the importance of evaluating diabetes prevention using both continuous biomarkers and categorical glycemic outcomes, particularly in longitudinal studies where opposing individual trajectories may coexist.

The clinical relevance of these findings extends beyond diabetes prevention, as improvements in glycemic status are associated with meaningful reductions in long-term cardiometabolic risk. Recent work highlights that normalization of glycemia may be particularly advantageous. Prediabetes itself represents a high-risk metabolic state associated with elevated long-term risks of atherosclerotic cardiovascular disease, heart failure, chronic kidney disease, and mortality, with more than 75% of this excess risk persisting independently of progression to diabetes (6). Although sustained normoglycemia may confer the greatest long-term protection, prior studies demonstrate that even transient regression to normoglycemia is clinically meaningful, being associated with reduced risks of T2D (23); microvascular disease (22); cardiovascular events (24, 25); and cardiovascular and all-cause mortality (25, 26). Thus, while glycemic normalization offers important benefits, transient improvements in glycemic status may nonetheless contribute to meaningful risk reduction as well.

Participants achieved sustained, clinically meaningful weight loss at 5 years, coinciding with favorable long-term metabolic outcomes. Mean weight loss at 5 years was 5.8 kg (5.3%), with nearly half reaching ≥5% loss, almost one-third achieving ≥10%, and 13% achieving ≥15%. This degree of long-term weight loss maintenance contrasts with patterns observed in many lifestyle interventions, where regain commonly occurs after the initial intervention period. For example, in the DPP, participants in the lifestyle arm maintained approximately 2% weight loss at 5 years (20), whereas participants in the present CCI sustained >5% loss on average after achieving greater reductions at one and two years. Importantly, sustained weight loss of ≥5% has been associated with delayed onset of cardiometabolic conditions in individuals with obesity, reductions in cardiovascular events in T2D, and lower cardiovascular morbidity and mortality in individuals with prediabetes (with evidence suggesting that benefits in the latter group are mediated through improvements in glycemic status) (24, 26–28). It is also notable that the degree of long-term weight loss in the present study was achieved at a time before the availability of newer pharmacotherapies that are now associated with substantially greater average weight loss.

The clinical significance of these outcomes is multifactorial, as weight loss has been shown to be a strong predictor of reduced diabetes incidence (29) and long-term metabolic success (30). Importantly, because the CCI emphasized glycemic regulation and carbohydrate reduction rather than calorie counting or weight targets, sustained weight loss in this cohort likely reflects downstream metabolic improvements rather than being the sole driver of benefit. Consistent with this interpretation, a prior analysis of individuals with prediabetes demonstrated that greater adherence to carbohydrate reduction (reflected by higher circulating ketone levels) was associated with normalization of fasting glucose, supporting a mechanism whereby improvements in glycemic control are linked to dietary carbohydrate reduction rather than weight loss alone (31). This mechanistic pathway may therefore contextualize the higher rates of reversion to normoglycemia observed in the present study. This interpretation is further supported by evidence that glycemic normalization can occur independently of weight loss in interventions involving carbohydrate reduction or intensive lifestyle change (32, 33).

In addition to changes in glycemic status and body weight, participants experienced improvements in several cardiometabolic risk markers at 5 years. Triglycerides, HDL-C, non–HDL-C, and LDL-C, as well as hsCRP, were generally maintained at or below baseline levels over 5 years, although these changes did not remain statistically significant after adjustment for multiple comparisons. In contrast, white blood cell count decreased significantly over time. Overall the pattern suggests relative stability across multiple domains relevant to cardiovascular-kidney-metabolic (CKM) health, including dysglycemia, atherogenic dyslipidemia, and systemic inflammation (34). These findings align with evidence linking low-carbohydrate dietary patterns to improvements in lipid and inflammatory markers that are associated with reduced risk of T2D (35, 36), including sustained reductions in triglycerides, which has been identified as an independent, dose-dependent risk factor for diabetes (37). Liver and kidney markers remained within normal limits throughout the study, supporting the long-term safety of this nutrition therapy within a monitored individualized clinical care model.

### 4.1 Strengths and limitations

Strengths of this study include its five-year duration, high retention (47% of the original cohort), breadth of cardiometabolic outcomes, and the real-world applicability of the digital care model. In addition, average BHB levels are consistent with dietary adherence through year three, and suggest sustained, though potentially partial or intermittent, adherence, to the low carbohydrate nutrition therapy in years four and five, during which time BHB targets were adjusted to each participant's personal health needs, values, and goals. Future studies could further examine health outcomes in relation to sustained nutritional ketosis, and varying levels of adherence over an extended time period. Limitations include the single-arm design, modest sample size, predominance of female participants, limited racial diversity, and potential selection bias among participants who agreed to the three-year extension study. Further randomized and comparative effectiveness studies would elucidate potential mechanisms and predictors of long-term success.

### 4.2 Conclusions

Participants in this study experienced improved markers of insulin resistance, clinically meaningful weight loss, and favorable cardiometabolic health profiles over time. Although mean glycemic markers were comparable to baseline at 5 years, these findings reflect heterogeneity in individual trajectories, with a substantial proportion of participants achieving normoglycemia and relatively few progressing to T2D. In conclusion, this study provides five-year evidence suggesting that a continuous remote care model featuring individualized carbohydrate-reduced nutrition therapy may support long-term diabetes prevention.

## Data Availability

The data analyzed in this study is subject to the following licenses/restrictions: data is available upon appropriate request to the corresponding author, accompanied by a detailed proposal outlining how the data will be used.

## In memorium

We honor the memory of Dr. Sarah J. Hallberg, whose leadership and contributions were invaluable to this research and included conceptualization, methodology, investigation, data curation, resources, supervision, and validation.
