# Supplementary material for: Five-Year outcomes of a digitally delivered carbohydrate-reduced nutrition intervention for prediabetes: durability of diabetes prevention
Source: Front Nutr. 2026 Jun 19;13:1839029. doi: 10.3389/fnut.2026.1839029 (PMC13329102; doi:10.3389/fnut.2026.1839029)
Supplement: Supplementary file 1 [file Data_Sheet_1.docx]

Supplementary Table 1. Sensitivity analysis of selected cardiometabolic outcomes using all observations and log-transformed variables (N=58).

|  |  | Baseline | |  | 1 year | |  | 2 years | |  | 3.5 years | |  | 5 years | |
| --- | --- | --- | --- | --- | --- | --- | --- | --- | --- | --- | --- | --- | --- | --- | --- |
| Fasting Insulin, mU/L† |  | 24.6 | (3.0) |  | 13.7 | (1.2)*** |  | 14.4 | (1.2)** |  | 16.3 | (1.3)** |  | 18.2 | (1.9)* |
| Fasting Insulin, ln |  | 3.0 | (0.1) |  | 2.4 | (0.1)*** |  | 2.5 | (0.1)*** |  | 2.6 | (0.1)*** |  | 2.7 | (0.1)** |
| HOMA-IR† |  | 7.2 | (1.2) |  | 3.2 | (0.3)** |  | 3.7 | (0.4)** |  | - | - |  | 5.0 | (0.5)* |
| HOMA-IR ln |  | 1.7 | (0.1) |  | 1.0 | (0.1)*** |  | 1.1 | (0.1)*** |  | - | - |  | 1.4 | (0.1)** |
| Triglycerides, mg/dL† |  | 158.5 | (11.2) |  | 121.6 | (9.7)** |  | 113.5 | (8.1)*** |  | 136.9 | (13.9) |  | 147.1 | (16.8) |
| Triglycerides, ln |  | 4.9 | (0.1) |  | 4.7 | (0.1)*** |  | 4.6 | (0.1)*** |  | 4.7 | (0.1)** |  | 4.8 | (0.1)* |
| hsCRP, mg/L† |  | 6.8 | (0.7) |  | 5.5 | (0.9)* |  | 5.0 | (0.8)** |  | - | - |  | 6.8 | (1.9) |
| hsCRP, ln |  | 1.6 | (0.1) |  | 1.2 | (0.1)*** |  | 1.0 | (0.1)*** |  | - | - |  | 1.3 | (0.1)* |
| Creatinine, mg/dL† |  | 0.9 | (0.0) |  | 0.8 | (0.0)*** |  | 0.8 | (0.0)* |  | - | - |  | 0.8 | (0.1) |
| Creatinine, ln |  | -0.2 | (0.0) |  | -0.2 | (0.0)*** |  | -0.2 | (0.0)** |  | - | - |  | -0.2 | (0.0) |
| AST, IU/L† |  | 24.2 | (2.3) |  | 20.9 | (1.1)* |  | 20.3 | (1.0) |  | - | - |  | 24.0 | (1.6) |
| AST, ln |  | 3.1 | (0.1) |  | 3.0 | (0.0) |  | 3.0 | (0.0) |  | - | - |  | 3.1 | (0.1) |
| ALT, IU/L† |  | 28.8 | (2.9) |  | 21.7 | (1.0)** |  | 21.9 | (1.2)* |  | - | - |  | 30.0 | (3.1) |
| ALT, ln |  | 3.2 | (0.1) |  | 3.0 | (0.0)** |  | 3.0 | (0.1)** |  | - | - |  | 3.2 | (0.1) |

Note. Values are mean (SE). Estimated means and standard errors were derived from linear mixed-effects models using maximum likelihood approach with an unstructured covariance matrix. Covariates included: baseline age, sex, race, and metformin use. This table includes only variables that demonstrated non-normal distributions and required sensitivity analysis. Results are shown for both models including all observations (without exclusion of the top 1% of values) and models using log-transformed variables. Ln log-transformed. All p-values are compared to baseline. * p<0.05, ** p<0.01, *** p<0.001. Abbreviations: HOMA-IR, homeostatic model assessment for insulin resistance; hsCRP, Highly Sensitive C-Reactive Protein; AST, Aspartate Aminotransferase; ALT, Alanine Aminotransferase

Supplementary Table 2. Glycemic and weight outcomes at 5 years among completers (N=45).

|  |  | Baseline (SE) |  | 5 years (SE) |  | Change from baseline (SE) |
| --- | --- | --- | --- | --- | --- | --- |
| Hemoglobin A1c (%) |  | 5.9 (0.0)) |  | 5.9 (0.1) |  | -0.05 (0.06) |
| Fasting glucose (mg/dL) |  | 110.9 (2.3) |  | 107.0 (2.5) |  | -4.0 (2.5) |
| Fasting insulin (mIU/L) |  | 21.7 (1.7) |  | 16.6 (1.6) |  | -5.1 (2.0) * |
| HOMA-IR (insulin-derived) |  | 5.9 (0.5) |  | 4.5 (0.5) |  | -1.4 (0.6) * |
| Weight (kg) |  | 109.7 (3.6) |  | 102.9 (3.2) |  | -6.8 (1.7) *** |

**Note**. Estimated means and standard errors were derived from linear mixed effect models using a maximum likelihood approach with the following covariates: baseline age, sex, race, and metformin use. *p<0.05, ***p<0.001 **Abbreviations**. HOMA-IR, homeostatic model assessment for insulin resistance

Supplementary Table 3. Two-year clinical characteristics difference between those consented versus those declined to long-term follow-up.

| Clinical Characteristics |  | Consented to 5-year Extension (N=58) |  | Declined 5-year Extension  (N=14) |  | P-value |
| --- | --- | --- | --- | --- | --- | --- |
| Weight (kg) |  | 96.0 (21.1) |  | 97.5 (21.3) |  | NS |
| HbA1c (%) |  | 5.7 (0.3) |  | 6.0 (0.2) |  | ** |
| Fasting glucose (mg/dL) |  | 100.9 (13.2) |  | 110.3 (18.3) |  | NS |
| Fasting insulin (mIU/L) |  | 14.7 (9.5) |  | 13.7 (9.8) |  | NS |
| Triglycerides (mg/dL) |  | 114.5 (61.0) |  | 98.9 (23.1) |  | NS |
| Total cholesterol (mg/dL) |  | 215.4 (50.0) |  | 198.9 (17.2) |  | NS |
| LDL-cholesterol (mg/dL) |  | 136.4 (44.3) |  | 128.4 (14.3) |  | NS |
| HDL-cholesterol (mg/dL) |  | 58.2 (18.2) |  | 52.6 (9.9) |  | NS |
| Non-HDL-cholesterol (mg/dL) |  | 157.3 (50.5) |  | 146.3 (17.2) |  | NS |
| Apolipoprotein B (mg/dL) |  | 107.4 (30.5) |  | 98.0 (7.7) |  | NS |
| Apolipoprotein A1 (mg/dL) |  | 173.0 (34.6) |  | 163.0 (27.3) |  | NS |
| hs C-reactive protein (nmol/L) |  | 5.1 (6.5) |  | 6.6 (9.2) |  | NS |
| White blood cell (kcumm) |  | 6.4 (1.8) |  | 7.3 (1.9) |  | NS |
| ALT (U/L) |  | 21.9 (8.8) |  | 20.0 (4.3) |  | NS |
| AST (U/L) |  | 20.4 (5.4) |  | 17.4 (1.6) |  | ** |
| ALP (U/L) |  | 68.4 (16.6) |  | 61.9 (14.6) |  | NS |
| Bilirubin (mg/dL) |  | 0.5 (0.2) |  | 0.5 (0.1) |  | NS |
| BUN (mg/dL) |  | 16.5 (5.5) |  | 15.0 (3.0) |  | NS |
| Creatinine (mg/dL) |  | 0.8 (0.3) |  | 0.8 (0.1) |  | NS |
| eGFR (mL/min/1.73m^2^) |  | 81.9 (13.0) |  | 83.6 (7.2) |  | NS |
| Beta-hydroxybutyrate (mM) |  | 0.2 (0.2) |  | 0.1 (0.1) |  | ** |

**Note.** Values are mean (SD) or percentage. **p<0.01. **Abbreviations:** NS, not significant; HbA1c, Hemoglobin A1c; LDL, low density lipoprotein; HDL, high density lipoprotein; ALT, alanine aminotransferase; AST, aspartate aminotransferase; ALP, alkaline phosphatase; BUN, blood urea nitrogen; eGFR, estimated glomerular filtration rate

Supplementary Table 4. Baseline characteristics between individuals who completed five years of follow-up and those lost to follow-up between years two and five.

|  |  | 5-Year Completers (n=45) |  | Non-Completers (n=13) |  | P-value |
| --- | --- | --- | --- | --- | --- | --- |
| Demographics |  |  |  |  |  |  |
| Age (years) |  | 53.8 (7.5) |  | 52.2 (11.0) |  | NS |
| Female (%) |  | 82 |  | 85 |  | NS |
| African American (%) |  | 2 |  | 15 |  | NS |
| Clinical Characteristics |  |  |  |  |  |  |
| Weight (kg) |  | 109.7 (25.6) |  | 108.3 (18.3) |  | NS |
| Hemoglobin A1c (%) |  | 5.9 (0.2) |  | 6.0 (0.2) |  | NS |
| Fasting glucose (mg/dL) |  | 111.0 (17.5) |  | 114.0 (14.4) |  | NS |
| Fasting insulin (mIU/L) |  | 25.7 (26.7) |  | 24.1 (13.0) |  | NS |
| HOMA-IR |  | 7.5 (10.4) |  | 6.9 (3.9) |  | NS |
| Triglycerides (mg/dL) |  | 162.3 (91.1) |  | 148.1 (62.9) |  | NS |
| Total cholesterol (mg/dL) |  | 209.6 (35.8) |  | 184.5 (20.6) |  | ** |
| LDL cholesterol (mg/dL) |  | 129.9 (35.2) |  | 107.5 (19.3) |  | ** |
| HDL cholesterol (mg/dL) |  | 50.8 (15.1) |  | 50.6 (11.8) |  | NS |
| Non-HDL cholesterol (mg/dL) |  | 158.9 (38.3) |  | 133.8 (25.4) |  | * |
| Apolipoprotein B (mg/dL) |  | 111.1 (25.9) |  | 94.8 (17.3) |  | * |
| Apolipoprotein A1 (mg/dL) |  | 161.4 (24.5) |  | 168.9 (25.0) |  | NS |
| hs C-reactive protein (nmol/L) |  | 6.3 (5.0) |  | 8.9 (8.0) |  | NS |
| White blood cell (kcumm) |  | 6.9 (1.9) |  | 7.3 (1.6) |  | NS |
| ALT (U/L) |  | 27.0 (16.4) |  | 35.1 (35.9) |  | NS |
| AST (U/L) |  | 24.8 (19.8) |  | 21.9 (10.5) |  | NS |
| ALP (U/L) |  | 72.6 (20.1) |  | 76.7 (19.4) |  | NS |
| Bilirubin (mg/dL) |  | 0.5 (0.3) |  | 0.5 (0.2) |  | NS |
| Blood Urea Nitrogen (mg/dL) |  | 16.7 (4.5) |  | 14.8 (4.6) |  | NS |
| Creatinine (mg/dL) |  | 0.88 (0.21) |  | 0.81 (0.21) |  | NS |
| eGFR (mL/min/1.73m2) |  | 77.9 (13.7) |  | 84.9 (13.6) |  | NS |
| Beta-hydroxybutyrate (mM) |  | 0.1 (0.2) |  | 0.1 (0.1) |  | NS |

**Note.** Values are mean (SD) or percentage. *p<0.05, **p<0.01. **Abbreviations:** NS, not significant; HOMA-IR, homeostatic model assessment for insulin resistance; LDL, low density lipoprotein; HDL, high density lipoprotein; hs, highly sensitive; ALT, alanine aminotransferase; AST, aspartate aminotransferase; ALP, Alkaline phosphatase; eGFR, Estimated glomerular filtration rate
